# Enzymatic Esterification of Functional Lipids for Specialty Fats: 1,3-Dipalmitoylglycerol and 1,3-Distearoylglycerol

**DOI:** 10.3390/molecules30061328

**Published:** 2025-03-16

**Authors:** Yuhuang Yang, Juanjuan Chi, Shengyuan Wang, Abdelaziz Elbarbary, Yafei Zhang, Jun Jin

**Affiliations:** 1 State Key Laboratory of Food Science and Resources, School of Food Science and Technology, Jiangnan University, Wuxi 214122, China; 2 Wilmar (Shanghai) Biotechnology Research & Development Center Co., Ltd., Shanghai 200137, China; 3 Dairy Science Department, Faculty of Agriculture, Benha University, Moshtohor 13736, Egypt; 4 Food Laboratory of Zhongyuan, Luohe 462300, China

**Keywords:** 1,3-dipalmitoylglycerol, 1,3-distearoylglycerol, enzymatic esterification, fat crystal, functional lipids

## Abstract

High-melting point 1,3-diacylglycerols not only provide health benefits, but are also suitable for manufacture of foods containing various specialty fats. It is difficult to prepare such high-melting point diacylglycerols, as the activities of specific enzymes will severely reduce at their melting points. In the present study, a combined technique was developed to prepare 1,3-dipalmitoylglycerol (1,3-DPG) and 1,3-distearoylglycerol (1,3-DSG) using selective esterification, molecular distillation, and solvent fractionation. Lipozyme TL IM was suitable for use as the optimal enzyme to maintain relatively high activity levels at esterification temperatures of 73–75 °C. 1,3-DAG/(DAG + TAG) was selected as the most important index to monitor the esterification and to evaluate the synthesized fats. The obtained 1,3-DPG and 1,3-DSG showed high purities, at more than 83%, and possessed hard attributes at room temperature. Both 1,3-DPG and 1,3-DSG exhibited fat crystals with β′ and β crystals. Needle-like and rod-like crystals were observed at 5–25 °C for 1,3-DPG, and closely packed feather-like crystals were found at 5–20 °C for 1,3-DSG, indicating their multiple abilities in modifying the crystallization stabilization of the fat matrix during food processing.

## 1. Introduction

Diacylglycerols (DAGs) have attracted significant research interest as their potential effects on lowering blood lipids have come to light in recent years [1,2]. Metabolism of these tends to generate energy metabolism products, with less being converted into fats for storage. The digestion and absorption process for diacylglycerols in the gastrointestinal tract is relatively slow, which leads to the transmission of satiety signals, giving people a stronger sense of satiety [3]. It is necessary to point out that such digestion and absorption behaviors of DAGs will not cause vitamin malabsorption and lead to nutritional deficiency problems [4,5]. Furthermore, the influence of DAGs on lipid crystallization is of great significance for foods containing fats and oils [6,7]. Taking cocoa butter as an example, the crystallization characteristics of DAGs may affect its crystallization and final quality of chocolates [8,9]. In particular, long carbon chain-saturated fatty acid DAGs, namely 1,3-dipalmitoylglycerol (1,3-DPG) and 1,3-distearoylglycerol (1,3-DSG), may have significant impacts on the positive effects of chocolates and chocolate products, such as improvement of heat stability and bloom-resistance abilities [10,11,12].

As 1,3-diacylglycerols (1,3-DAGs) are less common in natural fats and oils, enzymatic synthesis is a necessary and efficient technique to prepare 1,3-DAGs [13]. Specific enzymes are generally required to catalyze the generation of 1,3-DAGs in a solvent system or a solvent-free system, such as an esterification reaction between glycerol and fatty acids, a glycerolysis reaction between glycerol and triglycerides, or a transesterification reaction between triglycerides and DAGs [13,14]. After completing the enzymatic preparation, molecular distillation is the suggested method for removing extra fatty acids and the produced monoglycerides. To improve the concentration of 1,3-DAGs in the final synthetic products, solvent fractionation is performed to remove 1,2-DAGs and 2,3-DAGs [15].

It is a challenge to synthesize 1,3-DPG and 1,3-DSG in a solvent-free system using 1,3-sepcific enzymes as the melting points of palmitic acids and stearic acids are generally higher than the optimum activity temperatures of enzymes. With this in mind, an efficient system for synthesizing lipases should be developed by selecting suitable enzymes, optimizing reaction temperature and time, and removing produced water with absorbents and/or a vacuum environment. In the present study, a combinatorial purification technology, mainly comprising enzymatic esterification, molecular distillation, and solvent fractionation, was studied to obtain purified 1,3-DPG and 1,3-DSG, providing technical references for industrial production of 1,3-DAGs with high-melting point saturated fatty acids.

## 2. Results and Discussion

### 2.1. Gram-Scale Enzymatic Esterification of 1,3-DAGs

Lipozyme TL IM proved to be the optimal enzyme in this reaction system in the preliminary experiment. Among the four dehydrating agents, silica gel and the 4A molecular sieve showed relatively better effects, but these could still be improved, which will be discussed in the following sections. It was further suggested that the glycerol be mixed with silica gel and the 4A molecular sieve in equal amounts based on the preliminary experiment.

**Figure 1:**
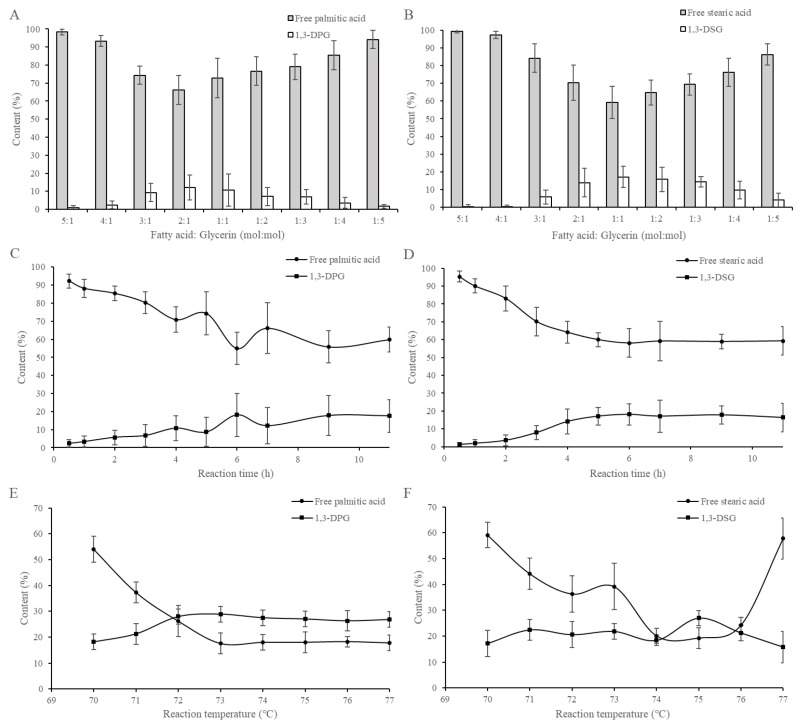
Enzymatic esterification conditions of 1,3-DPG (**A**,**C**,**E**) and 1,3-DSG (**B**,**D**,**F**).

The molar ratios of fatty acid to glycerin were studied from 5:1 to 1:5, as shown in Figure 1A,B. For the enzymatic esterification of 1,3-DPG (Figure 1A), when the molar ratio of palmitic acid to glycerol was 2:1, the lowest residual content of free palmitic acid was observed in the esterified product, accompanied by the highest content of 1,3-DPG, reaching 10%. In the case of enzymatic esterification of 1,3-DSG (Figure 1B), when the molar ratio of stearic acid to glycerol was 1:1, the residual stearic acid reached the lowest level, while the content of the produced 1,3-DAG was maximized, at nearly 20%. Further optimizations, mainly of reaction time and temperature, were carried out based on these reactant molar ratios.

Time courses of the enzymatic esterification are shown in Figure 1C,D. It was found that when the reaction time reached 6 h, the products exhibited the lowest amount of residual fatty acids, as well as the highest 1,3-DPG level, at nearly 20% (Figure 1C). In a similar case, the optimal time for the preparation of 1,3-DSG was also 6 h, reaching nearly 20% (Figure 1D).

Temperature courses of the enzymatic esterification are presented in Figure 1E,F. For the preparation of 1,3-DPG, when the reaction temperature was 73 °C, the esterified product demonstrated the lowest content of free palmitic acid, along with the highest content of 1,3-DPG, reaching 30%. In the case of preparation of 1,3-DSG, the content of free stearic acid was the lowest at a reaction temperature of 75 °C, while the 1,3-DSG level reached the highest, with nearly 30%.

### 2.2. Pilot-Scale Enzymatic Esterification of 1,3-DAGs

Based on the optimal esterification parameters, pilot-scale reactions were conducted in a 3 L double-layer glass reaction kettle under an absolute pressure of 0.01 MPa. For the enzymatic esterification of 1,3-DPG, the mass ratio of 4A molecular sieve, silica gel, and glycerol was set at 1:1:1, with a molar ratio of palmitic acid to glycerol of 2:1. The reaction temperature was controlled by a circulating water bath at 73 °C, and the reaction time was 6 h with a stirring paddle rotation speed of 275 rpm. As presented in Table 1, the crude products contained 35.04% DAGs, 74.98% of which was the 1,3-iosmer. There were still 30.53% free fatty acids (FFAs), 22.64% triglycerides (TAGs) and 11.79% monoglycerides (MAGs), which will be removed in the following purification process.

For the preparation of 1,3-DSG, the mass ratio of the 4A molecular sieve, silica gel, and glycerol was also 1:1:1, and the molar ratio of stearic acid to glycerol was 1:1. The esterification was conducted at 75 °C for 6 h, and other operation conditions were the same as those for preparing 1,3-DPG. There were 39.58% DAGs, 25.64% TAGs, 26.22% MAGs, and 8.56% FFAs. The ratio of 1,3-DAG to DAG was 52.43%.

### 2.3. Purification of Esterified DAGs Using Multi-Stage Molecular Distillation

Three-stage molecular distillation was further optimized to remove TAGs, MAGs, and FFAs in the crude esterified DAGs. In the first stage-treatment, FFAs and MAGs were designed to be removed at 170–190 °C. As shown in Table 1, only 1.59–1.85% FFAs were still detected in both residues, indicating that most of the FFAs were removed at this stage. However, although most of the MAGs were successfully distilled for the 1,3-DPG sample, the MAGs still accounted for 12.22% in the 1,3-DSG sample.

In the second step, higher evaporation surface temperatures, i.e., 220–230 °C, were applied to collect the DAGs from the first stage-residues. Most of the TAGs were removed at this stage. Only 5.65% TAGs were detected in the 1,3-DPG distillate, and 6.47% TAGs were found in the 1,3-DSG distillate.

A third stage-molecular distillation was then conducted to remove residual FFAs and MAGs in the second stage-distillates. The operation conditions were the same as those of the first stage-molecular distillation. The obtained 1,3-DPG residue contained 86.05% DAGs, whose 1,3-isomer accounted for 67.89%. Similarly, the 1,3-DSG residue comprised 63.11%, and the 1,3-isomer accounted for 68.55%. Compared with Wang et al. and Yeoh et al. [16,17], who obtained more than 80% DAG by using the two-step molecular distillation method, the target product in this paper has a higher purity, especially with the 1,3-DAG as the main DAG.

### 2.4. Solvent Fractionation for Concentrated 1,3-DAGs

Fractionation using hexane, which has the best solubility with non-polar substances, was further carried out to improve the proportions of 1,3-DAGs. At the optimal crystallization temperature, 25 °C, hexane was capable of partitioning 1,2-DAGs and some TAGs into the oleins, while the target 1,3-DAGs entered into the stearins. As presented in Table 1, 88.92% DAGs were detected in the 1,3-DPG stearin, and the ratio of 1,3-DPG to DAG was as high as 79.05%. The 1,3-DSG stearin contained 83.77% DAGs, and 62.28% of the DAGs comprised the 1,3-isomer. It was indicated that high 1,3-DAG proportions contributed to lowering lipid deposition and controlling obesity, as confirmed by both animal experiments and clinical research [18,19,20].

### 2.5. Solid Fat Contents of 1,3-DAGs

**Figure 2:**
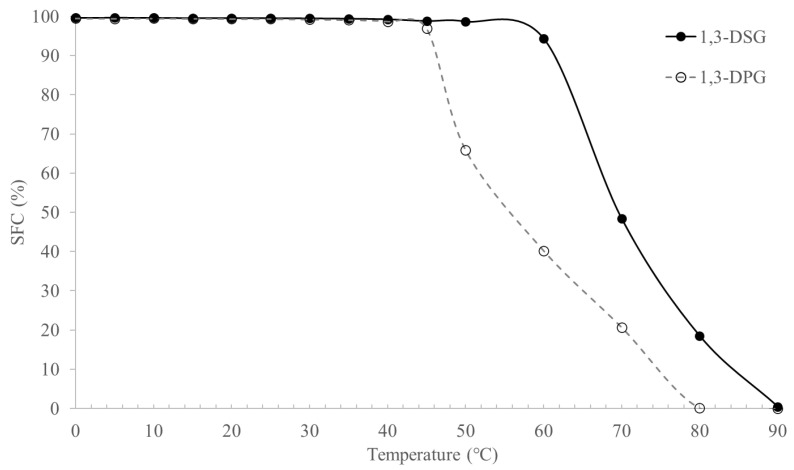
Solid fats contents of 1,3-DPG and 1,3-DSG.

SFC is an important parameter to evaluate potential usages of fats, as it reflects fat melting and crystallization behaviors, as well as related textural properties of final food products, such as plasticity and ductility [21,22]. As shown in Figure 2, both 1,3-DPG and 1,3-DSG exhibited nearly 100% solid fats at room temperature and body temperature. The profiles tended to decline at temperatures higher than 45 °C for DPG and 60 °C for DSG. It was suggested that such hard fats could be blended with low-melting fats and oils to make food instead of being sued alone. For instance, the DAGs are suitable for the manufacturing of different types of shortenings due to their plasticity and emulsifying properties, especially fluid shortenings, shortening flakes and chips, and shortenings powder [23]. With fluid shortenings as an example, the hard DAGs could be evenly distributed in liquid oils, e.g., soybean oil, rapeseed oil, and corn oil, to obtain desirable viscosity and processing attributes. In other cases, shortening flakes exhibited melting points of 43–54 °C for icing stabilizers and 60–66 °C for hard emulsifiers. Such fats require steep SFC profiles that were similar to those of the hard DAGs in the present study.

### 2.6. Fat Crystal Morphologies of 1,3-DAGs

**Figure 3:**
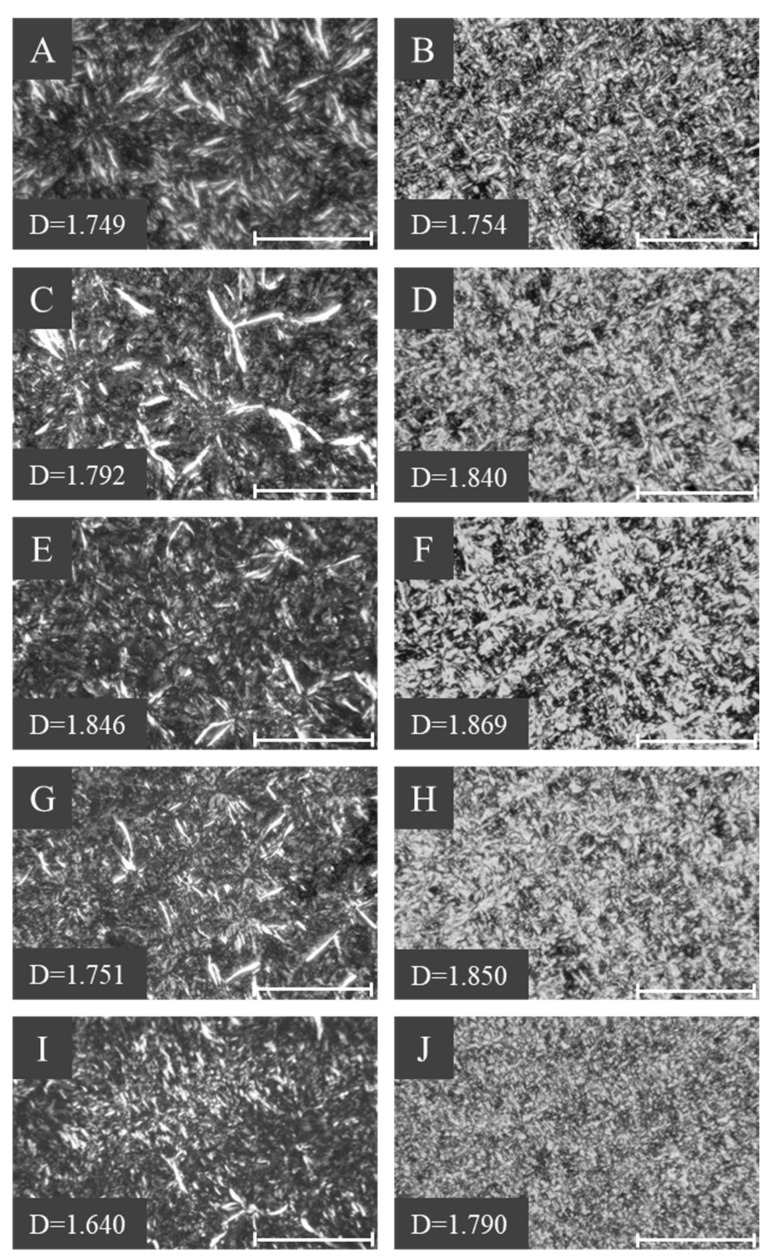
Fat crystals of 1,3-DPG and 1,3-DSG at 5 °C (**A**,**B**), 10 °C (**C**,**D**), 15 °C (**E**,**F**), 20 °C (**G**,**H**), and 25 °C (**I**,**J**) for 1 h (scale bar indicated 100 μm).

**Figure 4:**
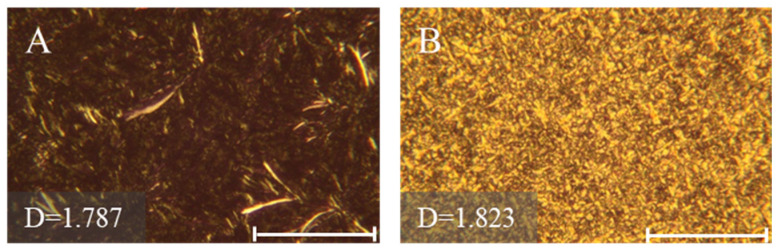
Stabilized fat crystals of 1,3-DPG (**A**) and 1,3-DSG (**B**) incubated from 20 to 25 °C (scale bar indicated 100 μm).

Fat crystals of DPG and DSG were observed from a polarized light microscope, and their morphologies, together with fractal dimensions, are presented in Figure 3 and Figure 4. The fractal dimensions is a quantitative measure that could be used to evaluate the spatial distribution of fat crystals, such as crystal size, shape, and packing [24]. 1,3-DPG exhibited needle-like, rod-like, and densely packed crystals, becoming radiating clusters (Figure 3A,C,E,G,I), with scattered distributions of feather-like crystals. The highest fractal dimension was observed at 15 °C (Figure 3E), indicating a greater degree of order in the packing. Its morphology remained the same after stabilizing from 20 to 25 °C, as shown in Figure 4A. Feather-like crystals with overlapping and close packing dominated in the 1,3-DSG samples crystallized at 5–20 °C (Figure 3B,D,F,H), while fine crystals were distributed densely at 25 °C (Figure 3J). In general, the needle-like and rod-like fat crystals contributed to accelerating the whipping progress and improving foam stabilities of whipped creams [24,25], while some feather-like crystals may improve melting properties for chocolate products [26]. For instance, feather-like crystals are usually observed in well-tempered chocolates. Closely packed granular and lamellar crystals were further found when the 1,3-DSG was stabilized from 20 to 25 °C (Figure 4B), which was close to the morphology obtained from 25 °C crystallization.

### 2.7. Fat Crystal Types of 1,3-DAGs

**Figure 5:**
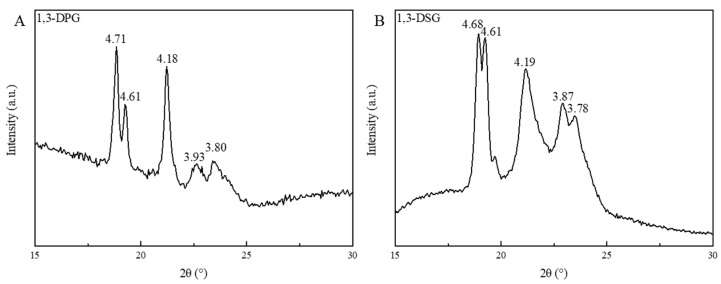
Polymorphism of 1,3-DPG (**A**) and 1,3-DSG (**B**) crystallized at a programmed temperature from 20 to 25 °C.

Polymorphism of 1,3-DPG and 1,3-DSG was further analyzed by X-ray diffractometer. As reported by D’Souza, Deman, and Deman [27] and Chen, Ghazani, Stobbs, and Marangoni [28], two strong short spacings at nearly 3.80 and 4.20 Å (or short spacings at 4.35, 4.15, 3.97, and 3.81 Å) represents β′ crystals, a very strong short spacing at nearly 4.60 Å and several weak spacings at nearly 3.98, 3.87, 3.75, and 3.67 Å are correspond to β_2_ crystal, and a prominent short spacing at nearly 4.60 Å and several weak spacings at 3.86 and 3.70 Å indicate β_1_ crystal. It could be found in Figure 5 that both 1,3-DPG and 1,3-DSG exhibited complex fat crystals with β_1_, β_2_ and β′ crystals, indicating their diverse abilities in modifying and controlling the crystallization stabilization of a fat matrix during food processing. For instance, β′ crystals are preferred in typical shortenings.

## 3. Materials and Methods

### 3.1. Materials

Palmitic acid (melting point: 63 °C), stearic acid (melting point: 69 °C), and silica gel (200–300 mesh) were purchased from Sinopharm Group Co., Ltd. (Shanghai, China). Lipozyme TL IM enzyme (immobilized carrier: maltodextrin) was purchased from Beijing Cliscent Technology Co., Ltd. (Beijing, China). The 4A molecular sieve was purchased from Shanghai Titan Scientific Co., Ltd. (Shanghai, China), AB-8 resin was purchased from Meryer Chemical Technology Co., Ltd. (Shanghai, China), and acrylic resin was bought from Shanghai Macklin Biochemical Co., Ltd. (Shanghai, China). Standards of triglycerides, diglycerides, and monoglycerides were purchased from Sigma-Aldrich Corporation (Shanghai, China). Other analytical and organic solvents were also purchased from Sinopharm Group Co., Ltd. (Shanghai, China).

### 3.2. Enzymatic Esterification of 1,3-DPG and 1,3-DSG

**Figure 6:**
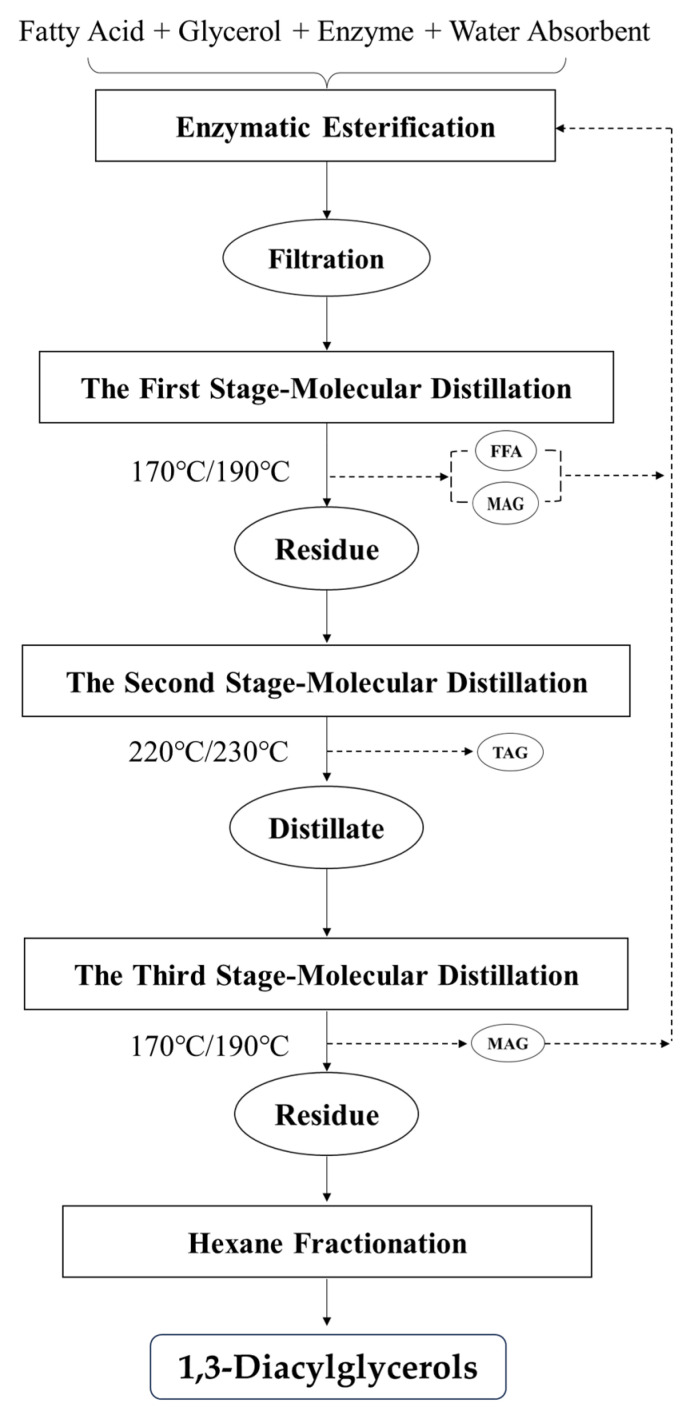
Technical protocol for the syntheses and purification of high-melting 1,3-DAGs.

The technical protocol for the syntheses and purification of 1,3-DAGs is shown in Figure 6. Dehydration condensation reactions for the enzymatic syntheses of 1,3-DPG and 1,3-DSG in a solvent-free system were carried out with a 1,3-specific lipase load of 15% in the present study.

During the reaction, the generated water vapor will be absorbed when passing through polar immobilized carriers of lipases, resulting in the enzymes binding to each other, as well as expanding and agglomerating into a mass, making it difficult to continue the reaction [29,30,31]. In this regard, water absorbent substrates were required to remove the water, and to prevent the carriers from absorbing water. The potential dehydrating agents, namely, silica gel, the 4A molecular sieve, AB-8 resin, and acrylic resin, were selected based on the reaction efficiencies. Also, the generated water was removed through pressure reduction (0.01 MPa). Such a vacuum environment showed inhibitory effects on acyl migration.

A single-factor experimental method was applied to optimize the key reaction parameters, mainly the molar ratio of fatty acid and glycerin (i.e., from 5:1 to 1:5), time (i.e., from 0.5 to 11 h) and temperature (i.e., from 70 to 77 °C), according to contents of free fatty acids and 1,3-DAGs in the synthesized products.

### 3.3. Purification of Esterified 1,3-DAGs Using Multi-Stage Molecular Distillation

The crude products of esterified 1,3-DAGs were purified using a molecular distillation apparatus (AYAN-F150 Molecular Distillation Apparatus, Shanghai, China), carried out through the method reported by Wang et al., and Yeoh et al. [16,17]. To obtain purified 1,3-DAGs, three-stage molecular distillation was carried out as follows:

The first-stage molecular distillation was suggested to remove FFAs and MAGs simultaneously in the crude products at 170 °C or 190 °C for 1,3-DPG or 1,3-DSG, respectively. Both the feed temperature and condensation surface temperature were set at 70 °C, and the feed rate was 3 mL/min. The absolute pressure was 0.01 mbar.

It was difficult to achieve complete separation with a single distillation in this case. Therefore, a second-stage molecular distillation was further carried out to separate the DAGs from TAGs, in which the evaporation surface temperature was 220 °C for 1,3-DPG and 230 °C for 1,3-DSG. Both the feed temperature and condensation surface temperature were changed to 80 °C at this stage, and the other parameters were the same as those in the first-stage treatment.

A third-stage molecular distillation was also involved to further remove residual FFAs and MAGs in the distillate obtained from the second-stage treatment. The evaporation surface temperature, as well as other operation paraments, were the same as the first-stage molecular distillation. Contents of 1,3-DAGs, 1,2-DAGs, TAGs, MAGs, and FFAs were detected to evaluate the efficiency of the multi-stage molecular distillation.

### 3.4. Purification of 1,3-DAGs Using Solvent Fractionation

To further improve the concentrations of 1,3-DAGs, referring to the fractionation methods, different polar solvents were selected [32,33]. Through preliminary small-scale experiments, hexane fractionation was developed with the following optimal conditions: the weight-to-weight (*w*/*v*) ratios of the obtained residue to hexane was 1:40, and the mixture was heated to 60 °C to remove any fat crystals, followed by cooling to 25 °C and being kept at this temperature for 2 days. The stearin containing improved concentrations of 1,3-DAGs was then separated as the final samples.

### 3.5. Determination of Lipid Components

Lipid components, mainly TAGs, 1,3-DAGs, 1,2-DAGs, MAGs, and FFA, were determined by a high-performance liquid chromatograph (RID-HPLC, Waters Technologies Inc., Milford, MA, USA) equipped with a differential refractive index detector. The determination conditions were as follows: chromatographic column: Sepax HP-Silica column (5 μm, 4.6 × 250 mm); mobile phase: n-hexane–isopropanol–formic acid (volume ratio of 15:1:0.003); column temperature: 30 °C; injection volume: 20 μL; sample concentration: 20 mg/mL; flow rate: 1 mL/min.

### 3.6. Determination of Solid Fat Contents of 1,3-DAGs

The obtained 1,3-DPG and 1,3-DSG were heated to 80 °C to eliminate any fat crystal memory. Solid fat contents (SFCs) of each DAG were determined according to the AOCS Official Method Cd 16b-93. The sample (2–3 mL) was poured into the nuclear magnetic tube and tempered at 80 °C for 30 min; and then held at 0 °C for 90 min to allow complete crystallization [34]. The values were determined at temperatures ranging from 0 to 90 °C at 5 °C intervals by equilibrating the nuclear magnetic tubes at these temperatures for 30 min before measurement.

### 3.7. Observation of Fat Crystal Microstructures of 1,3-DAGs

Crystal microstructures of 1,3-DPG and 1,3-DSG were observed by a polarized light microscope (PLM) equipped with a spot idea camera (PL-180; Shangguang, Shanghai, China). Samples were stored at 5, 10, 15, 20, and 25 °C for 1h before observation, respectively. Stabilized samples were stored at 20 °C for 1 d, and further stored at 25 °C for 2 d. The obtained PLM images were analyzed using the box counting method in the Image J 1.36b software, referring to the method in the literature [35].

### 3.8. Determination of Polymorphism of 1,3-DAGs

Crystalline forms of 1,3-DPG and 1,3-DSG were detected using an X-ray diffractometer (D2 PHASER, Bruker Corporation, Billerica, MA, USA) equipped with Cu-Ka radiation and a Ni filter (k = 1.54184 Å), operating at 10 mA and 30 kV. Each sample was crystallized at 20 °C for 1 d, followed by being stabilized at 25 °C for 2 d. Scans from 15 ° to 35 ° were performed at a rate of 6.0°/min.

### 3.9. Statistical Analysis

All the experiments were carried out in at least triplicate. All data were expressed as means ± standard deviation (SD). Analysis of variance (ANOVA) was performed using the statistical analysis software (Version 19.0) in SPSS.

## 4. Conclusions

Functional specialty fats, 1,3-DPG and 1,3-DSG, were successfully prepared using enzymatic esterification and multi-stage purification. The processes could provide a reference for the preparation of high-melting point DAGs with functional 1,3-isomers. Both DAGs exhibited high SFCs at room temperature, and the SFC profiles tended to decline at temperatures higher than 45 °C for DPG and 60 °C for DSG. PLM further revealed that most of the 1,3-DPG crystallized into densely packed radiate clusters with needle-like and rod-like crystals. It was suggested that such fat crystals may contribute to improving whipping performance for whipped creams and non-dairy creams. 1,3-DSG formed closely packed feather-like crystals at 5–20 °C and fine crystals at 25 °C, which may improve melting behaviors for confectionery products. Both DAGs exhibited complex fat crystals with β_1_, β_2_, and β′ crystals, indicating that further uses must be developed for their plasticity, emulsifying properties, and health benefits.

## Figures and Tables

**Table 1:** Lipid compositions (g/100 g) of 1,3-DPG and 1,3-DSG during enzymatic synthesis, molecular distillation purification, and solvent fractionation.

Test Item		TAG	FFA	1,3-DAG	1,2-DAG	MAG	1,3-DAG/(DAG + TAG)	1,3-DAG/DAG	DAG/(DAG + TAG)
Esterified 1,3-DPG		22.64 ± 0.22	30.53 ± 0.31	26.27 ± 0.22	8.77 ± 0.02	11.79 ± 0.12	45.55 ± 1.03	74.98 ± 1.53	60.75 ± 1.32
Esterified 1,3-DSG		25.64 ± 0.25	8.56 ± 0.03	20.75 ± 0.22	18.83 ± 0.12	26.22 ± 0.29	31.82 ± 0.83	52.43 ± 1.10	60.68 ± 1.78
The 1st molecular distillation									
1,3-DPG	Residue	60.57 ± 1.82	1.59 ± 0.01	25.28 ± 0.60	11.55 ± 0.13	1.00 ± 0.00	25.96 ± 0.41	68.63 ± 1.33	37.82 ± 0.89
1,3-DSG	Residue	28.86 ± 0.50	1.85 ± 0.01	39.01 ± 0.67	18.06 ± 0.24	12.22 ± 0.11	45.40 ± 0.90	68.35 ± 1.45	66.42 ± 1.23
The 2nd molecular distillation									
1,3-DPG	Distillate	5.65 ± 0.10	10.01 ± 0.17	23.48 ± 0.51	13.70 ± 0.33	47.16 ± 0.93	54.82 ± 0.99	63.15 ± 1.51	86.80 ± 1.88
1,3-DSG	Distillate	6.47 ± 0.13	2.82 ± 0.07	14.12 ± 0.32	8.00 ± 0.17	68.60 ± 1.77	49.38 ± 0.71	63.82 ± 1.70	77.38 ± 1.88
The 3rd molecular distillation									
1,3-DPG	Residue	10.10 ± 0.18	0.43 ± 0.00	58.42 ± 1.11	27.63 ± 0.49	3.42 ± 0.07	60.76 ± 1.11	67.89 ± 1.57	89.50 ± 2.57
1,3-DSG	Residue	33.02 ± 0.64	1.30 ± 0.01	43.26 ± 0.88	19.85 ± 0.43	2.57 ± 0.05	45.00 ± 0.83	68.55 ± 1.47	65.65 ± 1.66
Hexane fractionation									
1,3-DPG	Stearin	6.66 ± 0.12	0.19 ± 0.00	70.29 ± 1.44	18.63 ± 0.37	4.24 ± 0.03	73.54 ± 1.45	79.05 ± 1.50	93.03 ± 2.17
1,3-DSG	Stearin	15.12 ± 0.37	0.18 ± 0.00	52.17 ± 1.16	31.60 ± 0.65	0.92 ± 0.01	52.76 ± 1.20	62.28 ± 1.22	84.71 ± 1.33

## Data Availability

The original contributions presented in this study are included in the article. Further inquiries can be directed to the corresponding author.

## Abbreviations

The following abbreviations are used in this manuscript:

1,3-DPG	1,3-dipalmitoylglycerol
1,3-DSG	1,3-distearoylglycerol
DAG	diacylglycerol
FFA	free fatty acid
MAG	monoglyceride
TAG	triglyceride
